# 5-Isopropyl­idene-1,3-dithiolo[4,5-*d*][1,3]dithiole-2-thione

**DOI:** 10.1107/S160053680901335X

**Published:** 2009-04-18

**Authors:** Masaaki Tomura, Yoshiro Yamashita

**Affiliations:** a Institute for Molecular Science, Myodaiji, Okazaki 444-8585, Japan; b Department of Electronic Chemistry, Interdisciplinary Graduate School of Science and Engineering, Tokyo Institute of Technology, Nagatsuta, Midori-ku, Yokohama 226-8502, Japan

## Abstract

The title compound, C_7_H_6_S_5_, contains a 5-yl­idene-1,3-dithiolo[4,5-*d*][1,3]dithiole-2-thione framework, which is an important synthetic precursor of multi-dimensional organic superconductors and conductors. The mol­ecular framework is planar with an r.m.s. deviation of 0.012 Å for the non-H atoms. In the crystal structure, mol­ecules are linked by short inter­molecular S⋯S inter­actions [3.501 (5) and 3.581 (4) Å], constructing a zigzag mol­ecular tape network along the *c* axis.

## Related literature

For general background, see: Williams *et al.* (1992 ▶); Ishiguro *et al.* (1998 ▶). For the synthesis of the title compound, see: Misaki *et al.* (1992 ▶). For related structures with a 5-yl­idene-1,3-dithiolo[4,5-*d*][1,3]dithiole-2-thione framework, see: Bryce *et al.* (2000 ▶); Hock *et al.* (2002 ▶); Beck *et al.* (2006 ▶). For bond-length data, see: Allen *et al.* (1987 ▶). For values of van der Waals radii, see: Bondi (1964 ▶). For a description of the Cambridge Structural Database, see: Allen (2002 ▶).
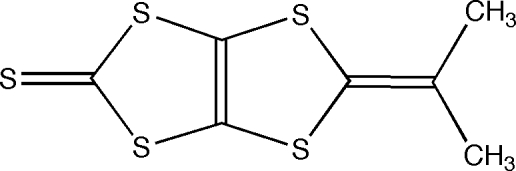


## Experimental

### 

#### Crystal data

C_7_H_6_S_5_
*M* *_r_* = 250.47
Triclinic, 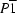

*a* = 7.082 (6) Å
*b* = 7.126 (6) Å
*c* = 10.534 (10) Å
α = 86.12 (3)°
β = 84.77 (3)°
γ = 71.95 (2)°
*V* = 502.9 (8) Å^3^
*Z* = 2
Mo *K*α radiation
μ = 1.09 mm^−1^
*T* = 291 K
0.09 × 0.02 × 0.01 mm

#### Data collection

Rigaku/MSC Mercury CCD diffractometer
Absorption correction: none
4550 measured reflections
2643 independent reflections
785 reflections with *I* > 2σ(*I*)
*R*_int_ = 0.117

#### Refinement

*R*[*F* ^2^ > 2σ(*F* ^2^)] = 0.072
*wR*(*F* ^2^) = 0.246
*S* = 0.84
2643 reflections
112 parameters
H-atom parameters constrained
Δρ_max_ = 0.46 e Å^−3^
Δρ_min_ = −0.50 e Å^−3^

### 

Data collection: *CrystalClear* (Rigaku/MSC, 2006 ▶); cell refinement: *CrystalClear*; data reduction: *TEXSAN* (Rigaku/MSC, 2004 ▶); program(s) used to solve structure: *SHELXS97* (Sheldrick, 2008 ▶); program(s) used to refine structure: *SHELXL97* (Sheldrick, 2008 ▶); molecular graphics: *PLATON* (Spek, 2009 ▶) and *Mercury* (Macrae *et al.*, 2006 ▶); software used to prepare material for publication: *SHELXL97*.

## Supplementary Material

Crystal structure: contains datablocks global, I. DOI: 10.1107/S160053680901335X/hg2498sup1.cif

Structure factors: contains datablocks I. DOI: 10.1107/S160053680901335X/hg2498Isup2.hkl

Additional supplementary materials:  crystallographic information; 3D view; checkCIF report

## Figures and Tables

**Fig. 1.**
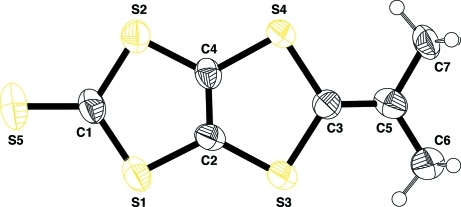
The molecular structure of (I), with atom labels and 50% probability displacement ellipsoids for non-H atoms and H atoms are shown as small spheres of arbitrary radii.

**Fig. 2.**
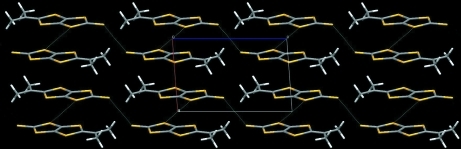
The packing diagram of (I), viewed along the b axis. Dashed lines indicate intermolecular S···S interactions.

## supplementary crystallographic information

### Comment

Molecules containing an 5-ylidene-[1,3]dithiolo[4,5-*d*][1,3]dithiole-2-thione framework are important synthetic precursors of multi-dimensional organic superconductors and conductors. Intermolecular S···S interactions involving peripheral sulfir atoms may increase the dimensionality in solid states and suppress metal-insulator transitions (Williams *et al.*, 1992; Ishiguro *et al.*, 1998). A search for the molecular framework in the Cambridge Structural Database (Version 5.30; Allen, 2002) gave only three examples (Bryce *et al.*, 2000; Hock *et al.*, 2002; Beck *et al.*, 2006). Thus, we report here the molecular and crystal structures of the title compound (I).

The compound (I) crystallizes in the *P*1 space group with one molecule in the asymmetric unit. The molecular structure is shown in Fig. 1. The bond lengths are within the normal ranges (Allen *et al.*, 1987). The molecular framework is planar with an r.m.s. deviation of 0.012 Å from the least-squares plane. In the crystal structure, the molecules are linked *via* short intermolecular S···S interactions [3.581 (4) for S1—S1(-*x*, -*y* + 3, -*z*) and 3.501 (5) Å for S5—S5(-*x*, -*y* + 3, -*z* - 1)] to construct a zigzag molecular tape network along the *c* axis (Fig. 2). The S···S interactions are 0.5–2.8% shorter than the sum of the corresponding van der Waals radii (Bondi, 1964). The molecules also form a π-stacking along the *a* axis with an interplanar distance of 3.54 (1) Å.

### Experimental

The title compound (I) was synthesized according to the literature method (Misaki *et al.*, 1992). Brown crystals of (I) suitable for X-ray analysis were grown from a dichloromethane solution.

### Refinement

All H atoms were placed in geometrically calculated positions and refined using a riding model, with C—H = 0.96 Å and *U*_iso_(H) = 1.5*U*_eq_(C).

### Crystal data

**Table:**

C_7_H_6_S_5_	*Z* = 2
*M**_r_* = 250.47	*F*(000) = 256
Triclinic, *P*1	*D*_x_ = 1.654 Mg m^−^^3^
Hall symbol: -P 1	Mo *K*α radiation, λ = 0.71070 Å
*a* = 7.082 (6) Å	Cell parameters from 843 reflections
*b* = 7.126 (6) Å	θ = 3.0–29.7°
*c* = 10.534 (10) Å	µ = 1.09 mm^−^^1^
α = 86.12 (3)°	*T* = 291 K
β = 84.77 (3)°	Needle, brown
γ = 71.95 (2)°	0.09 × 0.02 × 0.01 mm
*V* = 502.9 (8) Å^3^	

### Data collection

**Table:**

Rigaku/MSC Mercury CCD diffractometer	785 reflections with *I* > 2σ(*I*)
Radiation source: Rotating Anode	*R*_int_ = 0.117
Confocal	θ_max_ = 31.1°, θ_min_ = 3.0°
Detector resolution: 14.63 pixels mm^-1^	*h* = −9→9
φ and ω scans	*k* = −10→10
4550 measured reflections	*l* = −10→15
2643 independent reflections	

### Refinement

**Table:**

Refinement on *F*^2^	Secondary atom site location: difference Fourier map
Least-squares matrix: full	Hydrogen site location: inferred from neighbouring sites
*R*[*F*^2^ > 2σ(*F*^2^)] = 0.072	H-atom parameters constrained
*wR*(*F*^2^) = 0.246	*w* = 1/[σ^2^(*F*_o_^2^) + (0.0747*P*)^2^] where *P* = (*F*_o_^2^ + 2*F*_c_^2^)/3
*S* = 0.84	(Δ/σ)_max_ < 0.001
2643 reflections	Δρ_max_ = 0.46 e Å^−^^3^
112 parameters	Δρ_min_ = −0.50 e Å^−^^3^
0 restraints	Extinction correction: *SHELXL97* (Sheldrick, 2008), Fc^*^=kFc[1+0.001xFc^2^λ^3^/sin(2θ)]^-1/4^
Primary atom site location: structure-invariant direct methods	Extinction coefficient: 0.003 (3)

### Special details

**Table:**

Geometry. All e.s.d.'s (except the e.s.d. in the dihedral angle between two l.s. planes) are estimated using the full covariance matrix. The cell e.s.d.'s are taken into account individually in the estimation of e.s.d.'s in distances, angles and torsion angles; correlations between e.s.d.'s in cell parameters are only used when they are defined by crystal symmetry. An approximate (isotropic) treatment of cell e.s.d.'s is used for estimating e.s.d.'s involving l.s. planes.
Refinement. Refinement of *F*^2^ against ALL reflections. The weighted *R*-factor *wR* and goodness of fit *S* are based on *F*^2^, conventional *R*-factors *R* are based on *F*, with *F* set to zero for negative *F*^2^. The threshold expression of *F*^2^ > σ(*F*^2^) is used only for calculating *R*-factors(gt) *etc*. and is not relevant to the choice of reflections for refinement. *R*-factors based on *F*^2^ are statistically about twice as large as those based on *F*, and *R*- factors based on ALL data will be even larger.

### Fractional atomic coordinates and isotropic or equivalent isotropic displacement parameters (Å^2^)

**Table:**

	*x*	*y*	*z*	*U*_iso_*/*U*_eq_	
S1	0.1573 (3)	1.3556 (3)	−0.12379 (19)	0.0502 (6)	
S2	0.2864 (3)	0.9827 (3)	−0.25144 (18)	0.0496 (6)	
S3	0.1902 (3)	1.1364 (3)	0.13884 (18)	0.0452 (6)	
S4	0.3259 (3)	0.7524 (3)	0.00739 (18)	0.0434 (6)	
S5	0.1908 (4)	1.3598 (4)	−0.4066 (2)	0.0710 (9)	
C1	0.2089 (11)	1.2401 (12)	−0.2662 (7)	0.048 (2)	
C2	0.2080 (10)	1.1425 (11)	−0.0257 (7)	0.0427 (19)	
C3	0.2706 (10)	0.8745 (11)	0.1547 (7)	0.0407 (18)	
C4	0.2678 (9)	0.9729 (12)	−0.0871 (7)	0.0385 (17)	
C5	0.2896 (10)	0.7741 (12)	0.2681 (8)	0.045 (2)	
C6	0.2430 (11)	0.8811 (12)	0.3900 (7)	0.056 (2)	
H6A	0.3602	0.8472	0.4365	0.084*	
H6B	0.1395	0.8439	0.4405	0.084*	
H6C	0.1994	1.0210	0.3711	0.084*	
C7	0.3596 (11)	0.5529 (11)	0.2761 (7)	0.049 (2)	
H7A	0.3818	0.5036	0.1917	0.073*	
H7B	0.2603	0.5056	0.3239	0.073*	
H7C	0.4816	0.5078	0.3179	0.073*	

### Atomic displacement parameters (Å^2^)

**Table:**

	*U*^11^	*U*^22^	*U*^33^	*U*^12^	*U*^13^	*U*^23^
S1	0.0512 (13)	0.0409 (13)	0.0522 (14)	−0.0080 (10)	−0.0035 (10)	0.0126 (10)
S2	0.0551 (14)	0.0473 (14)	0.0417 (12)	−0.0113 (11)	0.0001 (9)	0.0038 (10)
S3	0.0519 (13)	0.0337 (12)	0.0451 (12)	−0.0077 (10)	0.0010 (9)	−0.0006 (9)
S4	0.0513 (13)	0.0308 (12)	0.0429 (12)	−0.0058 (10)	−0.0014 (9)	0.0009 (9)
S5	0.0751 (18)	0.078 (2)	0.0561 (16)	−0.0221 (15)	−0.0135 (12)	0.0300 (13)
C1	0.049 (5)	0.044 (5)	0.047 (5)	−0.007 (4)	−0.011 (4)	0.012 (4)
C2	0.046 (5)	0.031 (5)	0.047 (5)	−0.008 (4)	−0.002 (4)	−0.003 (4)
C3	0.040 (4)	0.039 (5)	0.040 (4)	−0.010 (4)	0.004 (3)	−0.001 (3)
C4	0.032 (4)	0.038 (4)	0.040 (4)	−0.004 (3)	−0.006 (3)	0.006 (3)
C5	0.039 (4)	0.037 (5)	0.058 (5)	−0.014 (4)	0.013 (4)	−0.001 (4)
C6	0.066 (6)	0.050 (6)	0.051 (5)	−0.016 (5)	0.002 (4)	−0.005 (4)
C7	0.056 (5)	0.039 (5)	0.049 (5)	−0.016 (4)	0.002 (4)	0.014 (4)

### Geometric parameters (Å, °)

**Table:**

S1—C1	1.716 (8)	C3—C5	1.346 (10)
S1—C2	1.738 (8)	C5—C6	1.496 (10)
S2—C4	1.723 (7)	C5—C7	1.497 (10)
S2—C1	1.744 (8)	C6—H6A	0.9600
S3—C2	1.725 (8)	C6—H6B	0.9600
S3—C3	1.775 (8)	C6—H6C	0.9600
S4—C4	1.758 (7)	C7—H7A	0.9600
S4—C3	1.783 (7)	C7—H7B	0.9600
S5—C1	1.652 (7)	C7—H7C	0.9600
C2—C4	1.341 (10)		
			
S1···S1^i^	3.581 (4)	S5···S5^ii^	3.501 (5)
			
C1—S1—C2	96.7 (4)	C3—C5—C6	120.7 (8)
C4—S2—C1	94.9 (4)	C3—C5—C7	121.2 (7)
C2—S3—C3	94.4 (3)	C6—C5—C7	118.1 (7)
C4—S4—C3	94.3 (4)	C5—C6—H6A	109.5
S5—C1—S1	123.5 (5)	C5—C6—H6B	109.5
S5—C1—S2	122.1 (5)	H6A—C6—H6B	109.5
S1—C1—S2	114.4 (4)	C5—C6—H6C	109.5
C4—C2—S3	119.7 (6)	H6A—C6—H6C	109.5
C4—C2—S1	115.0 (6)	H6B—C6—H6C	109.5
S3—C2—S1	125.3 (5)	C5—C7—H7A	109.5
C5—C3—S3	123.3 (6)	C5—C7—H7B	109.5
C5—C3—S4	122.0 (6)	H7A—C7—H7B	109.5
S3—C3—S4	114.6 (4)	C5—C7—H7C	109.5
C2—C4—S2	118.9 (6)	H7A—C7—H7C	109.5
C2—C4—S4	117.0 (6)	H7B—C7—H7C	109.5
S2—C4—S4	124.1 (5)		
			
C2—S1—C1—S5	180.0 (5)	S3—C2—C4—S2	−179.5 (4)
C2—S1—C1—S2	−0.9 (5)	S1—C2—C4—S2	−0.4 (8)
C4—S2—C1—S5	179.9 (5)	S3—C2—C4—S4	−0.3 (8)
C4—S2—C1—S1	0.8 (5)	S1—C2—C4—S4	178.8 (3)
C3—S3—C2—C4	0.3 (6)	C1—S2—C4—C2	−0.2 (6)
C3—S3—C2—S1	−178.7 (5)	C1—S2—C4—S4	−179.3 (5)
C1—S1—C2—C4	0.8 (6)	C3—S4—C4—C2	0.1 (6)
C1—S1—C2—S3	179.8 (5)	C3—S4—C4—S2	179.2 (4)
C2—S3—C3—C5	179.6 (7)	S3—C3—C5—C6	−0.2 (10)
C2—S3—C3—S4	−0.2 (4)	S4—C3—C5—C6	179.7 (5)
C4—S4—C3—C5	−179.8 (7)	S3—C3—C5—C7	−179.5 (5)
C4—S4—C3—S3	0.1 (4)	S4—C3—C5—C7	0.3 (10)

Symmetry codes: (i) −*x*, −*y*+3, −*z*; (ii) −*x*, −*y*+3, −*z*−1.
